# Supplementary material for: Are carbon emissions trading and green financial instruments synergistic? -Comprehensive quantitative research based on content analysis
Source: PLoS One. 2024 Mar 7;19(3):e0298601. doi: 10.1371/journal.pone.0298601 (PMC10919601; doi:10.1371/journal.pone.0298601)
Supplement: S1 Data — (ZIP) [file pone.0298601.s001.zip › Data/地级市Z_F工作报告.zip!/░▓╗╒/│╪╓▌╩╨/└┤╫╘╣1⁄2╓┌║┼í╛┬φ┐╦╩2╛▌═°í┐_2015.docx]

各位代表：

现在，我代表市人民政府，向大会作工作报告，请予审议，并请市政协委员和其他列席人员提出意见。

一、2014年工作回顾

过去的一年，面对复杂多变的宏观环境和艰巨繁重的改革发展稳定任务，全市人民在省委、省政府和市委的坚强领导下，深入贯彻落实党的十八大、十八届三中、四中全会和习近平总书记系列重要讲话精神，主动适应经济发展新常态，创新思路，转变方法，统筹做好稳增长、促改革、调结构、惠民生、防风险的各项工作，经济社会发展保持了稳中有进、提质增效的良好态势。初步测算，全市生产总值突破500亿元，增长9.2%左右；财政收入92亿元，增长10.2%；固定资产投资540亿元，增长17%；社会消费品零售总额161.2亿元，增长13.5%；进出口总额4.4亿美元，增长7.3%；城镇居民人均可支配收入增长9%，农村居民人均可支配收入增长12%。

一是坚持加快转型升级，工业经济结构不断优化。紧紧围绕主导产业定位，在转型升级中做大总量。规模工业增加值166.3亿元，增长12.5%，增幅居全省第三；新增规模以上工业企业79户；工业化率38.5%，同比提升0.6个百分点。

进一步明晰园区特色定位。制定实施省级以上开发园区产业发展指导目录，推动工业园区集中、“两高一首”产业集聚、科技创新集成、转型发展集效，全市园区完成投入370亿元，主导产业集中度82%，对全市规模工业的贡献率77.7%，同比分别提高2个、1.3个百分点。江南产业集中区产业承载功能持续完善，新引进落户项目90个，其中投产26个，完成固定资产投资71.5亿元、增长18.2%，实现财政收入4亿元。市开发区列入首批国家低碳工业园区试点。县区工业园区产业竞争力进一步增强，前江工业园区销售收入率先突破百亿元。

大力培育战略性新兴产业。坚持特色发展、错位发展，装备制造、生物医药、新材料、新能源等战略性新兴产业实现产值97亿元，增长18.6%；高新技术产业产值240亿元，增长30%，增幅居全省第一。电子信息产业新引进项目27个，建成投产11个，实现产值70亿元、增长18%，初步形成芯片设计制造、封装测试、整机及电子基础材料全产业链。强化科技创新驱动，新增国家级技术创新示范企业1家、省级高新技术企业14家、企业技术研发机构8家，新认定国家重点新产品2个，荣获省科技一等奖项目1个，列入全省高层次科技人才创新创业团队1个，新增中国驰名商标1件、省著名商标16件、省名牌产品5个，青阳县列入国家知识产权强县工程试点县，东至县被评为全省发明专利授权量“十强县”。

多措并举增强企业活力。坚持分类施策、精准用力，扎实开展政策落实“专项集中推进行动”，综合运用政银企对接合作、市场重组、完善政策、依法处置等措施，18家企业招商重组盘活存量资产7.6亿元，结构性减税等减免缓企业资金7.2亿元，融资性担保公司在保余额40.3亿元、增长7.5%，为200户企业提供到期信贷周转资金15亿元，规模工业利润增长3.4%。增强金融服务和创新能力，交通银行池州分行获准设立，石台、东至扬子村镇银行开业运营，九华股份IPO预披露，7家企业在“新三板”、“四板”挂牌，企业在资本市场直接融资取得重大突破。全年社会融资总量125亿元，其中银行新增投放96亿元，中小微企业新增贷款39.5亿元、增长17.7%。

二是坚持发挥特色优势，旅游经济带动力不断增强。制定出台了贯彻落实皖南国际文化旅游示范区建设发展规划纲要的实施意见和推进商贸活市战略的政策措施，突出政府引导、市场主体，以游客需求为导向，统筹推进旅游项目、景区创建、宣传营销、服务配套以及旅游环线建设，接待国内外游客4133.3万人次、增长20.1%，旅游收入421.9亿元、增长22.3%，服务业增加值185亿元、增长8.2%。

着力完善旅游综合交通体系。攻坚克难构建快速便捷的大交通格局，对外打开通道，内部畅通循环，池州长江公路大桥开工建设，望东长江公路大桥完成投资过半，宁安城际池州段完成轨道铺设，东九高速主体工程完工，济祁高速连接线秋浦河大桥建成使用，国省干线公路新开工19.2公里、建成27.1公里，完成交通投资32.8亿元。4条过江通道等一批重点交通项目列入长江经济带综合立体交通走廊规划。

全面推进旅游核心区建设。坚持引进市场主体增加旅游产品供给，列入省“861”计划的40个文化旅游招商项目完成投资18.9亿元。杏花村文化旅游区起步区生态本底建设基本完成，签约引进项目14个、总投资148.5亿元，8个项目开工建设。九华山文明燃香长效机制基本建立，大愿文化园接待游客47.7万人次。新增4A级景区3个，四星级以上农家乐17家。举办旅游营销推介活动60余场（次），与3家境外旅游集团建立合作机制，九华山机场旅客吞吐量22.2万人次，池州港接待长江游轮163航次、游客13.2万人次。平天湖入选央视十佳“中秋最美赏月地”。

持续加强旅游服务业态融合。坚持推进旅游与体育、文化、商务融合发展，利用市场化运作方式举办了第四届全国绿运会、第六届世界传统武术锦标赛、2014中国会展业年会等重大赛事展会，引进落户大型电子商务企业5家、总部经济项目3个、现代物流项目18个以及设计、咨询等中介服务机构8家，石台县列入全国首批电子商务进农村综合示范县，青年（大学生）创业园获批省电子商务示范园区，新增限上批发零售和住宿餐饮业单位67家、亿元流通企业2家。

三是坚持推进统筹发展，城乡一体化进程不断加快。实施创新生态文明建设体制机制的若干意见，突出以人的城镇化为核心，统筹推进人居城市、新型城镇化、美好乡村“三大建设”，创建省级生态市通过考核验收，市辖三县完成国家级生态县创建目标任务，列入全省水生态文明建设第一批试点城市，生态宜居的城市特色进一步彰显。

切实抓好“三农”工作。加快发展现代农业，创建省级现代农业示范区1个，粮食总产68万吨，新建标准化蔬菜基地1000亩，新改建无性系良种茶园1万亩，3家茶企进入全国行业百强，新增“三品”认证11个；有序推进农村土地承包经营权确权登记颁证试点，土地流转7万亩，新增家庭农场等新型农业经营主体275个、规模农产品加工企业18家。着力改善农村基础设施条件，解决15.96万农村人口饮水安全问题，实施中小河流治理工程5处、排涝泵站工程2个、小型水库除险加固19座，完成水利投资6.1亿元；改造县乡道路157.3公里、农村公路危桥93座；升级改造农村电网1300公里；建成高标准基本农田18.1万亩，土地整理新增耕地7125.6亩。兑现强农惠农政策补贴资金6.5亿元，农民人均收入水平不断提高。

着力提升中心城市功能。97个市本级政府性投资项目完成投资9.1亿元，新改建城市道路18.1公里、雨污管网29.5公里、燃气管线96公里，地下综合管线完成普查。制定平天湖和清溪河水环境综合治理规划，完成清溪河主河道清淤、生态驳岸、尾水改造及沿线22个溢流口、2条沟渠截污整治，清溪河水质明显改善，论证确定平天湖为主城区应急备用水源。编制实施国家绿色生态示范城区规划，天堂湖环境综合整治进展顺利。“数字池州”公共云平台建成使用，市容管理不断加强。主城区房地产市场健康稳定发展。

稳步推进新型城镇化。有序推进农业转移人口市民化，全面推行居住证制度，进城落户农民纳入城镇住房和社会保障体系。扎实开展县城规划建设管理水平提质达标行动，重点推进东至、青阳省级新型城镇化试点，完成县城及集镇改造投资7.8亿元，新开通城乡公交线路6条。

扎实推进美好乡村建设。按照“三美”标准，坚持因地制宜、突出特色，尊重群众意愿，整合资源要素，引导农民参与，以“四沿五片”为重点推进“三线三边”环境治理，加快改善农村人居环境，实施10个乡镇农村清洁工程，改造农村危房6048户，建成25个省级重点示范村，贵池区在全省考核中跻身“美好乡村建设先进县”。

深入推进生态文明建设。强化以大气污染防治为重点的环境保护，可吸入颗粒物平均浓度59微克/立方米，下降幅度居全省第一，空气质量居全省第二，人居环境在全省城市居民幸福指数分析评价中位居第一。节能减排完成省定目标。认真实施千万亩森林增长工程，植树造林8.27万亩，治理水土流失18.3平方公里。完成矿山环境综合整治21家，整顿关闭非煤矿山36家，完成地质灾害点避让搬迁治理4处。依法、科技治超和整治非法采砂的力度进一步加大。依法清理闲置低效用地2500亩。

四是坚持深化改革创新，市场主体活力不断迸发。突出转变政府职能，加快重点领域和关键环节改革，规范政府行为，公平配置资源，优化市场环境，招商引进规模项目460个，建成投产253个，亿元以上省外项目到位资金294亿元，实际利用外资3亿美元；吸引民间投资390亿元，增长20%，占总投资的73%，民营经济增加值占全市经济比重达到64%。

加大简政放权力度。坚持以政府权力的“减法”换取市场活力和社会创造力的“加法”，大力推进行政审批制度改革，市级行政审批事项减少53%，建设项目联合审批的平均办理时限减少50%，缓征、降标、取消行政事业性收费16项，取消建设项目前置中介收费3项。基本建立涉企收费清单制度，精简收费项目24.2%。全面实施注册资本认缴登记制改革，净增私营企业1895户、个体工商户5655户，分别增长20.6%、10.4%。兑现支持民营经济发展资金1.05亿元，2家民企进入全省企业百强。工商、质监、食品药品监管体制改革基本完成，港航管理体制改革、事业单位分类改革稳步推进。

推进公共资源管理创新。制定出台政府向社会力量购买服务的实施意见和目录，采取政府和社会资本合作模式（PPP），在全国首批试点实施污水处理及市政排水设施购买服务项目。制定出台行政事业单位国有资产管理办法，政府性资金和债务全口径预算管理体系不断健全，市属投融资公司法人治理结构进一步完善，公共资源配置基本实现标准程序公开、市场竞争取得、结果公平公正。

五是坚持保障改善民生，社会建设和治理水平不断提升。坚持保基本、兜底线，扎实推进基本公共服务体系建设，民生类支出111亿元，增长9%，占财政总支出的80%。

着力提高民生工程质效。建立完善投入多元化、内容公开化、管理绩效化机制，33项民生工程和10件惠民实事投入资金34.2亿元，发放医疗、孤儿、贫困生、贫困残疾人、生活无着人员等各类补助救助资金13.6亿元，工程项目后续管养投入资金6600万元，群众满意率居全省前列。

加强就业和社会保障体系建设。创业就业技能培训1.23万人，新增城镇就业1.8万人，转移农村劳动力1.87万人，城镇登记失业率3.4%。企业退休人员养老金人均月增161.6元，“老字号”群体工龄补助全面发放，城镇居民基本医保和新农合大病保险启动实施。城乡低保实现动态管理、应保尽保，普惠制高龄老人津贴补助897.9万元，市老年公寓一期工程主体完工，改扩建敬老院8所，新增社会养老床位1140张。保障性住房开工10019套、基本建成5155套，棚户区改造11703户，发放廉租住房租赁补贴6657户，山区库区农村住房保险试点参保34.8万户。530户渔民全部上岸安居。建设工程农民工工资专户管理制度初步建立。36个村整村扶贫开发完成投入5217万元。

努力提升城乡基本公共服务水平。完成主城区学校布局规划编制，主城区新增中小学校2所，全市新建和改建乡镇公办幼儿园11所，建设义务教育标准化学校73所，规范办学行为专项治理深入开展。巩固县级公立医院综合改革成果，药品实现零差率销售，引进设立2家非公立二级综合医疗机构，建成市第三人民医院。新建1094个广播电视“村村通”工程，完成第三次经济普查和第一次可移动文物普查认定工作。“单独二孩”政策稳步实施，人口自然增长率4.29‰。妇女、儿童、老年人和残疾人事业加快发展，供销、气象、地震、民族、宗教、对台、外事、侨务、档案、保密、地方志、新闻出版、社会科学等各项工作不断加强。

不断创新社会治理。坚持多方参与、系统治理，主城区新创建标准化社区11个、全国和谐社区建设示范社区3个，第九届村委会换届选举工作顺利完成，70个农村社区试点启动建设，乡镇为民服务中心实现全覆盖，97%的村建成村级代理工作站。“六五”普法深入开展，法律援助2288件。安全生产形势持续稳定，食品安全、物价、市场秩序监管进一步加强。社会治安防控体系不断健全，群众安全感和满意度均居全省第一，信访秩序规范有序，科学预警防范和应急处置能力进一步提升。精神文明建设深入推进，9人入选“中国好人榜”、“安徽好人榜”，贵池区、东至县、青阳县成功创建安徽省文明县城（城区），石台县荣获安徽省创建文明县城工作先进县。国防动员、人民防空和民兵预备役等工作成效明显。

一年来，我们坚持“三严三实”要求，扎实开展党的群众路线教育实践活动，按照市委部署，认真落实“10+5”的重点整改方案，认真查找并积极整改“四风”方面存在的问题，政府系统作风建设进一步加强。坚持科学民主依法决策，坚持重大事项集体研究决定，新常态下防范处置金融、债务、经济运行等风险能力不断增强。自觉接受人大监督，依法执行人大决议决定，办理人大代表议案、建议163件。主动接受政协民主监督，办理政协委员提案344件，注意听取各民主党派、工商联和无党派人士的意见。深化政务公开和政府信息公开，回复办理市长信箱、市长热线、网民留言6024条，市及各县区政务服务群众满意度、信息公开工作连续两年在全省名列前茅。严格执行中央“八项规定”、国务院“约法三章”和省委“三十条”、市委“二十条”要求，严肃财经纪律，严控“三公”经费支出，健全廉政风险防控和处置长效机制，强化政府投资、经济责任、公共财政绩效等审计监督，加大效能建设和责任追究力度，自身建设取得新成效。

各位代表！过去的一年，面对经济持续下行的压力和各类要素约束的加大，在全国都面临着超乎寻常的挑战下，我们团结拼搏，砥砺奋进，取得了来之不易的成绩。这是省委、省政府和市委坚强领导的结果，是全市人民同心同德、顽强拼搏的结果，是我们主动适应、主动应对、主动作为，脚踏实地、艰苦奋斗的结果。在此，我代表市人民政府，向广大工人、农民、知识分子、干部、离退休老同志，向驻池解放军指战员、武警官兵和政法公安干警，向各民主党派、各人民团体和各界人士，向关心、支持池州发展的中央和省驻池单位，向在我市创业的投资者、建设者，向给予市政府高度信任和倾力支持的各位人大代表、政协委员，表示衷心的感谢！

回顾过去的一年，我们清醒地认识到，当前世界经济仍处在国际金融危机后的深度调整期，我国经济发展已进入速度变化、结构优化、动力转换的新常态，池州发展的环境、条件和内涵也发生了新的变化，新常态下的速度变化是必然趋势，结构调整伴随阵痛是不得不过的关口，前进道路上还有不少困难和问题。主要是：发展不足，发展不优，产业结构不尽合理，县域经济实力仍然较弱，经济基础还很脆弱；资源要素约束加剧，企业生产经营困难增多，投资增速持续回落，财政收入增速放缓，刚性支出不断增加，经济发展面临的形势仍较严峻，地区生产总值等指标实际完成情况与年初确定的计划有缺口；城乡居民收入持续较快增长难度加大，基本公共服务供给与需求还不适应，社会事业发展和社会治理创新需要进一步加强；政府职能转变任务艰巨，对微观经济干预过多与监管不到位的问题同时存在，运用老办法解决问题的思维惯性仍在延续，面对新常态，少数干部还存在着不敢为、不会为的现象。对此，我们要以对人民、对历史高度负责的态度，敢于担当，毫不懈怠，扎实有效地加以解决。

二、2015年工作安排

今年（2015）是全面深化改革的关键之年，是全面推进依法治市的开局之年，也是全面完成“十二五”规划的收官之年。我们要主动地适应新常态的“九大趋势性变化”，按照遵循经济规律的科学发展、遵循自然规律的可持续发展、遵循社会规律的包容性发展的要求，强化“五位一体”发展的整体性、系统性思维，积极抢抓长江经济带和“三区”发展的重大战略机遇，加快培育新的增长动力，努力推动经济社会实现更高质量、更可持续的发展。

今年（2015）政府工作的总体要求是：深入贯彻党的十八大、十八届三中、四中全会和习近平总书记系列重要讲话精神，主动适应经济发展新常态，坚持稳中求进总基调，坚持“三区”发展总思路，以生态文明建设为主线，以提高发展质量和效益为中心，突出全面深化改革、全面推进依法治市“两轮驱动”，强化工业、旅游“两大产业支撑”，推动转型升级，统筹城乡发展，注重民生改善，确保经济平稳健康发展、社会和谐稳定。

综合考虑，今年（2015）经济社会发展的主要目标是：全市生产总值550亿元、增长8.5%左右，财政收入突破100亿元、增长8.6%以上，固定资产投资增长13%，规模工业增加值增长12%以上，社会消费品零售总额增长13%，进出口总额增长10%，城镇居民人均可支配收入增长9%，农村居民人均可支配收入增长10%，城镇新增就业1.37万人，城镇登记失业率控制在4.5%以内，居民消费价格涨幅控制在3%左右，人口自然增长率控制在7‰以内，节能减排达到省控目标，单位生产总值建设用地降低率5%。

重点抓好以下六个方面工作：

（一）全力加快工业经济发展，在皖江城市带承接产业转移示范区建设中增强实力

坚持市场调节、进退并举、有扶有控，紧紧依靠市场配置资源、依靠创新驱动升级、依靠园区集群集效，进一步支持实体经济发展，做强工业经济，提升总体实力和竞争力。

加快培育新的经济增长点。紧盯消费市场，实施主导产业提升计划，分行业制定产业发展、技术升级和品牌培育路线图，选准重点突破的关键点和终端产品，引导传统产业通过招商重组和改造升级，加快技术、产品、管理和商业模式创新，向中高端持续迈进，重点推进80个重大工业项目建设，新增规模以上工业企业50户以上，主导产业产值比重提高2个百分点。以电子信息产业发展规划为引领，着力在龙头企业和产业链核心环节的招商上实施突破，引进中高端项目20个以上，建成投产10个以上，产值增长20%以上。

大力实施创新驱动战略。围绕产业链部署创新链，完善以人为本、企业主体、产学研用一体的技术创新体系，推动科技和产业深度融合，力争新增省级高新技术企业10家、国家级工程技术中心1家、省级企业技术研发机构8家，发明专利授权量增长15%以上，创建中国驰名商标1件、省著名商标10件、省名牌产品2个，战略性新兴产业产值、高新技术产业增加值增幅高于全省平均水平。健全人才引进市场化机制，加快培育一批创新团队、管理团队、资本运作团队。

强化产业平台支撑。继续推进园区基础设施提升和生活类服务设施配套，积极引导社会资本参与园区建设，加快产城一体融合发展。按照园区产业发展指导目录，加大招商合作力度，严格资源节约集约和环境准入门槛，大力开展低效闲置土地清理，力争省级以上园区各引进1个以上10亿元工业大项目，亩均投资强度和税收贡献分别提高2个百分点以上。重点推进江南产业集中区加强央企对接合作，集中精力招大引强，新引进2个以上20亿元工业大项目，完成工业投资50亿元，新增规模以上工业企业10户以上，实现财政收入4.5亿元。

促进工业经济平稳运行。认真落实中央和省、市促进经济平稳健康发展的政策措施，加强中小企业公共服务体系建设，完善产需衔接、银企对接、用工服务、产学研合作平台，打通政策落实“最后一公里”。注重运用市场化手段和差别化政策，依法依规，分类施策，鼓励企业兼并重组、靠大靠强，通过优化股权、改善管理走出困境。

（二）聚力提升以旅游为主导的现代服务业水平，在皖南国际文化旅游示范区建设中展示魅力

坚定不移地打好生态牌、九华牌、长江牌，充分彰显旅游发展优势，紧扣世界佛文化观光胜地和国际生态休闲城市目标定位，加快建设“两圈一带”，构建“一主三辅”扇形旅游集散体系，推进市、山、县（区）联动发展，进一步提升旅游经济竞争力和带动力，接待国内外游客4630万人次、旅游收入477亿元，分别增长12%、13%，服务业增加值增长8%以上。

推进旅游全域化发展。制定实施全域旅游发展规划，推动旅游与中心城市、城镇、美好乡村一体建设，与文化、生态、体育、会展、农业融合发展，加快20个5亿元以上项目建设，新建一批旅游停车场、自驾车营地，提升齐山-平天湖风景区、湿地森林公园旅游功能。发挥九华山的龙头带动和辐射作用，推进九华山申报世界地质公园、杏花村建设国家文化产业示范园区、升金湖创建国家湿地公园、牯牛降创建5A级景区等工作，新增4A级景区1家。扎实推进乡村旅游富民工程，新增省优秀旅游乡镇5个、旅游示范村6个、4星级以上农家乐8家。拓展航线市场和游轮市场，开展高铁沿线城市促销，推进九华山机场国际直航包机和航线申报，全年旅客吞吐量25万人次以上。加快发展智慧旅游，提高旅游消费便利化、信息化水平。深入推进旅游改革，推动旅游市场向社会资本全面开放，完善区域旅游合作机制。深入开展旅游市场秩序整治。

提升金融服务水平。坚持信贷投放与直接融资并重，引导实体经济与金融资本和社会资本有效对接，新增社会融资总量130亿元，其中银行新增投放90亿元以上。创新续贷、年审制贷款和循环贷款业务，缩短融资链条，确保涉农信贷投放总量持续增长，小微企业贷款增速不低于全部贷款增速、增量不低于上年。大力发展民营金融机构，推进国有融资性担保公司市场化参股运作，规范发展小额贷款公司和金融服务公司，引导发展互联网金融。确保九华股份主板上市，5家以上企业实现“新三板”、“四板”挂牌融资，力争发行一批中期票据、私募债、企业债。切实防范金融和债务风险。

加快发展现代服务业态。大力发展现代物流、科技、设计、信息等生产性服务业，积极引进第三方物流企业，加快发展制造业物流、商贸物流、电子商务物流。大力发展电子商务新业态，促进电商、网购、快递等融合发展，支持创建电子商务特色网络交易平台，引导企业、景区和创业者发展互联网营销。积极培育和引进会展主体，力争全年举办各类规模展会20个。积极引进国内外总部企业及分支机构，新增总部经济项目5个以上。

大力培育新的消费热点。加快发展居住休闲、健康养生、绿色运动等生态产业，实施养老健康家政、信息、旅游、住房、绿色、教育文化体育等领域消费工程，支持社会力量举办养老、健身、健康等服务机构，满足群众多层次多样化消费需求。鼓励新能源汽车、节能产品等绿色消费。认真落实带薪休假制度，促进旅游休闲消费。

（三）大力推动城乡统筹发展，在生态文明建设示范区建设中彰显优势

坚持把解决好“三农”问题作为重中之重，加快转变农业发展方式和农业结构调整，分层次推进人居城市、新型城镇化、美好乡村建设，进一步展现美丽池州新面貌。

加快推进农业现代化。稳定提升农业综合生产能力，扎实开展粮油棉高产创建活动，粮食总产稳定在66万吨以上；深入实施新一轮“菜篮子”工程，完善蔬菜基地产供销一体化体系；大力发展生态休闲观光农业，加快生态优质特色农产品开发，建设无性系茶叶良种基地1万亩，新增“三品”认证10个。加强农业农村基础设施建设，深入实施小型水利工程改造提升“5588”行动计划，开工建设3处中小河流治理工程，解决10万农村人口饮水安全问题，完成水利投资4亿元以上；升级改造县乡公路80公里，实施村级公路网化工程147公里，加固改造农村危桥40座；加快500千伏涓桥输变电工程等8项电网工程建设，升级改造农村电网730公里；建设高标准基本农田18万亩，耕地保有量保持在198.7万亩以上；加强气象防灾减灾体系建设，强化基层农技推广服务、重大动植物疫病防控和农产品质量安全监管。全面深化农村综合改革，大力培育新型农业经营主体，发展农业社会化服务，加快农村土地确权登记颁证，推进土地承包经营权流转健康发展，新增土地流转面积8万亩，发展家庭农场200家，争创省级以上农民专业合作社示范社5个。

不断彰显中心城市特色魅力。严格实施城市总体规划，划定城市开发边界，优化城市形态和空间格局，创新建设模式，完善城市功能，提升建设品质，让城市环境更宜居、生活更方便、发展机会更多。坚持政府投资保基本、市场投资多元化，健全政府和社会资本合作机制，畅通社会资本进入城市建设领域渠道，市本级安排政府性投资项目105个，其中市本级政府直接投资71个，年度计划投资15.5亿元，重点推进实施平天湖和清溪河水环境综合治理、路网及地下管网升级改造等城市功能提升工程。优先发展公共交通，完善城市慢行系统。推进城市精细化管理，建成社会治安视频监控、“数字城管”等智慧城市信息应用系统。加强市容市貌、交通秩序整治。进一步明确责任、权利、义务，加大依法整治力度，不断提升居住小区物业管理水平。促进房地产市场健康稳定发展。

扎实推进新型城镇化。推进“多规合一”试点，编制实施新型城镇化规划，加强城乡规划督察，以“三治三增三提升”为突破口，加快提升县城规划建设管理水平。积极盘活存量资产资源，吸引社会资本参与，完善城镇基础设施和公共服务设施，促进人口和产业集聚，着力打造特色县城和产业城镇。加快“三权落实”、“五有并轨”，有序推进户籍制度改革，促进教育、医疗、就业、住房保障等城镇基本公共服务常住人口全覆盖，引导农业转移人口有序向城镇转移。

持续推进美好乡村建设。坚持点上示范与普遍受惠相结合，发挥农民主体作用，加快建设第二批34个中心村，启动第三批38个中心村建设，统筹抓好农村道路、公共设施、居住环境等建设，因地制宜发展特色产业，壮大农村集体经济，建立健全长效管护机制。深入推进“三线三边”环境治理，加快垃圾污水、建筑、广告标牌、矿山治理和绿化提升，完成20家矿山环境治理和生态恢复。

大力推进生态文明建设。进一步完善生态文明建设体制机制，建立分类考核指标体系，实施差别化的区域开发和环境管理政策。争创国家生态市、国家环保模范城市，推进全国低碳城市、市开发区国家低碳工业园区和升金湖湿地生态效益补偿试点。加强环境保护、水土保持和生态修复，强化节能减排，全方位治理大气污染，严格控制各类环境污染。加强饮用水水源地保护，人工造林3.21万亩，整顿关闭非煤矿山13家，治理水土流失20平方公里。

（四）合力加强改革创新和法治建设，在充分激发市场主体活力中赢得先机

全面深化重点领域和关键环节改革，更好地发挥法治的引领和规范作用，创造既生机勃勃又井然有序的经济社会发展环境。

加快转变政府职能。纵深推进简政放权，继续做好国务院、省政府取消下放行政审批项目的衔接落实工作，做到应减尽减。全面实施政府权力清单、责任清单、涉企收费清单制度，加快建立负面清单制度。大力精简前置审批，规范中介服务，完善事中事后监管机制，制定市级投资项目审批核准前置要件目录，推行前置审批与项目核准并联办理，推进投资创业便利化。继续推进新一轮政府机构改革，加快事业单位分类改革，稳步推进其他各项改革。

大力加强法治建设。健全法治池州建设体制机制，完善法治池州建设考核评价体系。深化行政执法体制改革，推行食品药品安全、工商质监、公共卫生、安全生产、交通运输、城乡建设等领域综合执法，完善执法程序，规范行政裁量权标准，推进行政执法和刑事司法衔接。加快社会信用体系建设，探索建立违法信息共享、失信惩戒机制。坚持“谁执法、谁普法”，深入开展法治宣传教育，推进覆盖城乡的公共法律服务体系建设，引导全民自觉守法、遇事找法、解决问题靠法。

着力完善公平竞争的市场环境。强化国有资产、财政性资金、政府性债务全口径管理，推进公共资源配置、政府性投资项目建设、招投标交易全过程规范，确保标准公开、程序公开、结果公开，各类市场主体权利平等、机会平等、规则平等，保障市场主体和社会公众的知情权、参与权、监督权。落实职能部门法定权限和职责范围内的监管责任，全面实施企业信息公示制度，平等保护各类市场主体合法权益。

充分激活市场主体。全面落实支持民营经济发展政策措施，消除各种市场准入门槛，营造亲商、安商、富商浓厚氛围，让社会资本、人才、劳动力等各种市场要素在池州集聚，催生新的增长点。深入推进商事制度改革，加快青年大学生创业园等各类创业创新基地建设，形成大众创业、万众创新的社会氛围，净增私营企业1600户以上、个体工商户5600户以上。

（五）强力推进招商引资和项目建设，在持续扩大有效投入中增强后劲

坚持以开放的胸怀集聚资源要素，以务实的作风加快项目建设，不断增强经济发展后劲。

切实提升招商引资水平。制定主导产业招商导则，加大央企、知名民企、上市公司、行业龙头企业和金融招商力度，强化产业链上下游“补链、强链”招商，推进现代农业、城乡基础设施、非基本公共服务等领域全方位招商，积极支持存量资产重组招商。梳理完善新常态下的招商政策体系，健全以质量、效益和产业导向为主的绩效评估机制。坚持领导招商、行业协会招商、产业链招商、以商招商等行之有效的招商方式，规范务实高效地开展招商活动。全年招商引进项目470个，到位资金480亿元，建成投产300个；实际利用外资3.3亿美元。

着力推进项目建设提质提效。坚持领导联系重点项目和分级分类调度制度，落实项目工作责任，依法高效做好审批备案、征收安置、环境保障等工作，确保全年亿元以上项目开工80个，竣工45个。坚持政府投资做“减法”、市场投资做“加法”，在轨道交通、过江通道、矿石运输专线、一级道路等领域谋划一批能够由市场主体投资建设的重点推动性项目。结合“十三五”规划编制，谋划一批重大基础设施和重点领域产业项目，力争列入国家和省投资计划项目100个以上。

加快互联互通的开放大通道建设。对接长江经济带综合立体交通走廊规划，推进长江干支流航道整治，加强岸线资源保护开发利用和港口建设，加快建设畅通高效的黄金水道。做好宁安城际铁路通车准备，加快池州长江公路大桥等重大交通基础设施项目建设，推进黄山-九华山-天柱山旅游轻轨、六安-池州-景德镇铁路、梅龙过江通道等项目前期工作。

（六）着力保障改善民生和推进社会建设，在切实加强和创新社会治理中促进和谐

坚持民之所望为施政所向，计划投入35亿元，实施33项民生工程和10件惠民实事，重点解决好群众急需的基本民生问题，切实维护社会公平正义，加快共享改革发展新成果。

实施更加积极的就业政策。更好地发挥市场在促进就业中的作用，完善城乡均等的就业创业公共服务体系，结合产业升级开发更多优质岗位，加快形成创业带动就业良性机制，推进用工信息传递和岗位供需对接，组织就业技能培训0.9万人，农村劳动力转移就业1.8万人。依法维护劳动者合法权益。

完善社会保障体系。推进城乡养老保险关系有效接续转移，加强城乡低保信息化管理，提高农村五保、优抚对象保障水平，做好医疗救助、孤儿救助、临时救助、灾害救助工作，完善社会救助和保障标准与物价上涨挂钩联动机制。发展老龄事业，保障妇女、儿童和残疾人合法权益，支持红十字、慈善公益事业。新开工保障性安居工程12146套、基本建成10739套，发放廉租住房租赁补贴6644户。坚持科学扶贫、精准扶贫，增强贫困地区内生发展活力。

协调发展社会事业。实施第二期学前教育三年行动计划，推进义务教育均衡发展，建立规范办学常态化管理机制；深化产教融合，整合职教资源，加快培养产业发展急需的技能人才。继续深化县级公立医院改革，大力发展非公立医疗机构，推进基本公共卫生服务网格化管理，继续加强血防工作，巩固血防成果，抓好重大传染病、职业病防控。大力实施文化惠民工程，加强文物、非物质文化遗产保护和开发利用，积极申报和保护中国传统村落。以创建和美文化为抓手，深入开展群众性精神文明创建活动，启动全国文明城市争创工作。稳定适度的低生育水平，提高出生人口素质。推进全民健身，发展体育产业。扩大友城交流合作。夯实统计基层基础，加强和完善部门统计，提高源头数据质量。开展第二次全国地名普查。加强侨务、对台、供销、保密、档案、地方志、防震减灾、新闻出版、社科研究等各项工作。

夯实社会治理基层基础。推进城乡社区管理体制改革，实现主城区标准化社区全覆盖，有序实施农村社区建设试点，加强社区网格化管理和信息化建设，搭建社会管理服务综合平台，动员组织城乡居民有序参与社会治理。深化村务公开民主管理，推进乡村为民服务。支持社会组织发挥行业自律和专业服务功能，发挥工会、妇联、共青团等人民团体作用，大力倡导志愿服务。

健全公共安全体系。深入推进社会治安综合治理，严厉打击违法犯罪活动。健全依法维权和化解纠纷机制，加强人民调解、行政调解、司法调解衔接联动，推进依法信访。坚持重大事项社会稳定风险评估，加强网络舆情监测处置和正面引导，完善政府新闻发布制度，增强新媒体时代社会沟通能力。严格落实安全生产责任制，加强食品安全、产品质量、市场物价、建设工程质量监管，抓好超限超载治理、采砂整治、地质灾害防治等工作。加强应急管理，提高防灾减灾救灾和应对突发事件能力。依法维护民族团结和宗教和谐稳定。支持驻池部队建设，加强国防教育、国防动员、民兵预备役和人民防空工作，争创全国双拥模范城“四连冠”。

三、加强政府自身建设

推动发展转型，首先要加快政府转型；建设法治池州，首先要建设法治政府。必须全面加强政府自身建设，提高工作能力水平，促进治理体系和治理能力现代化。

（一）深入推进依法行政。坚持法无授权不可为、法定职责必须为，依法全面履行政府职能，不断提高运用法治思维和法治方式深化改革、推动发展、化解矛盾、维护稳定的能力。坚持科学民主依法决策，把公众参与、专家论证、风险评估、合法性审查、集体讨论决定作为重大行政决策法定程序，建立重大决策终身责任追究制度及责任倒查机制。全面落实行政执法责任制，严格规范公正文明执法。自觉接受同级人大及其常委会监督，依法执行人大决议决定，重大决策出台前向人大报告，认真办理人大代表议案、建议。主动接受政协民主监督，认真办理政协委员提案，诚恳听取各民主党派、工商联和无党派人士意见。更加注重推进政府信息公开，广泛接受人民群众监督。

（二）持续加强作风建设。坚持“三严三实”，巩固党的群众路线教育实践活动成果，认真抓好整改任务落实，坚决持续地治理“四风”问题。落实领导干部包片联户制度，深入基层，深入群众，加强调查研究。继续精简会议和文件，规范检查评比活动。扎实开展作风评议，加强行政监察，督促职能部门依法履职尽责，树立作风建设新形象，确保政务服务群众满意度继续保持在全省前列。

（三）严格落实工作责任。牢固树立机遇意识、责任意识、进取意识，坚持发展、主动作为，勇于负责、敢于担当。健全分管领导抓分管、主管部门抓行业、地方政府抓区域的责任体系，确保政令畅通、令行禁止。强化目标管理、督查推进、绩效考核、责任追究，坚持褒奖先进、鞭策后进，激励各级干部进一步提振精气神、凝聚正能量。

（四）保持清正廉洁形象。严格落实政府系统廉政主体责任，认真履行党风廉政建设责任制，用制度管权管事管人，把廉政建设落实到政府工作的每一个环节。认真落实中央“八项规定”、国务院“约法三章”和省委“三十条”、市委“二十条”要求，严格执行厉行节约反对浪费、公务接待管理等政策规定，压缩“三公”经费，推进公务用车改革。强化审计监督，推进公共资金、国有资产、国有资源、政府性投资项目和领导干部经济责任审计全覆盖。加强对政府班子自身和公务员队伍的教育、培训、管理和监督，进一步树立为民、务实、清廉的政府形象。

各位代表！全面完成今年（2015）发展目标，实现“十二五”圆满收官，根本在于理念转型，关键在于真转实干。我们要紧密团结在以习近平同志为总书记的党中央周围，在省委、省政府和市委的坚强领导下，进一步解放思想、奋发有为，锐意进取、扎实工作，不断开创“三区”发展新局面，为加快建设经济繁荣、环境优美、社会和谐、生活富裕的幸福池州而努力奋斗！
